# Ice Formation during PEM Fuel Cell Cold Start: Acceptable or Not?

**DOI:** 10.1002/advs.202302151

**Published:** 2023-06-21

**Authors:** Jinqiao Liang, Linhao Fan, Qing Du, Yan Yin, Kui Jiao

**Affiliations:** ^1^ State Key Laboratory of Engines Tianjin University 135 Yaguan Road Tianjin 300350 China; ^2^ National Industry‐Education Platform of Energy Storage Tianjin University 135 Yaguan Road Tianjin 300350 China

**Keywords:** cold start, degradation mechanism, ice formation, proton exchange membrane fuel cell

## Abstract

Proton exchange membrane (PEM) fuel cell faces the inevitable challenge of the cold start at a sub‐freezing temperature. Understanding the underlying degradation mechanisms in the cold start and developing a better starting strategy to achieve a quick startup with no degradation are essential for the wide application of PEM fuel cells. In this study, the comprehensive in situ non‐accelerated segmented techniques are developed to analyze the icing processes and obtain the degradation mechanisms under the conditions of freeze–thaw cycle, voltage reversal, and ice formation in different components of PEM fuel cells for different freezing time. A detailed degradation mechanism map in the cold start of PEM fuel cells is proposed to demonstrate how much degradation occurs under different conditions, whether the ice formation is acceptable under the actual operating conditions, and how to suppress the ice formation. Moreover, an ideal starting strategy is developed to achieve the cold start of PEM fuel cells without degradation. This map is highly valuable and useful for researchers to understand the underlying degradation mechanisms and develop the cold start strategy, thereby promoting the commercialization of PEM fuel cells.

## Graphical abstract

The degradation mechanisms under the conditions of freeze–thaw cycle, voltage reversal, and ice formation in different components of PEM fuel cells for different freezing time are revealed. The different degradation degrees and mechanisms under conditions are summarized into a degradation mechanism map, which can help researchers understand the underlying degradation mechanisms in various processes and develop the cold start strategy.

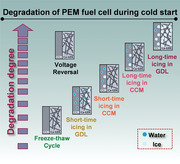

## 1 Introduction

During the transformation of global energy structure, emission‐free or nearly emission‐free electrification is essential in many fields, especially in transportation field.^[^ ^1^, ^2^, ^3^ ^]^ Proton exchange membrane (PEM) fuel cell can directly convert the chemical energy in hydrogen fuel into the electrical energy with water as the product, which is regarded as a promising power device.^[^ ^4^, ^5^, ^6^ ^]^ As same as the other power sources used in transportation, such as internal combustion engines and lithium batteries, PEM fuel cell also faces the challenge of starting at a sub‐freezing temperature.^[^ ^7^, ^8^, ^9^, ^10^ ^]^ Specially, the water as the only product will freeze during the cold start and thus highly affects the performance and durability of PEM fuel cell, which is the great limit of PEM fuel cell commercialization.^[^ ^11^, ^12^ ^]^

During the cold start, the water has several states, including vapor, liquid, super‐cooled water, ice, as well as frozen/non‐frozen membrane water, of which the phase change determines whether the cold start of PEM fuel cell can success.^[^ ^13^ ^]^ Under a sub‐freezing temperature, the ice formation might block the reactant gas paths and cover the catalyst sites, thereby leading to the failure of cold start and the degradation of PEM fuel cell.^[^ ^14^, ^15^, ^16^ ^]^ In addition, the inevitable freeze–thaw cycle and the possible voltage reversal during the cold start will also affect the durability of PEM fuel cell.^[^ ^17^, ^18^ ^]^ Nowadays, many countries and regions have put forward the specific standards on the durability of PEM fuel cell, such as the United States, Japan, and European Union.^[^ ^19^, ^20^, ^21^, ^22^ ^]^ Therefore, the experimental research on the degradation mechanisms of PEM fuel cell during the cold start, especially caused by ice formation, is essential to develop the cold start strategies to suppress degradation, thereby improving the durability of PEM fuel cell.

The degradation of PEM fuel cell includes the reversible degradation, which can be mitigated by recovery procedures, and the irreversible degradation, which should be avoided. The irreversible degradation is usually caused by the irreversible damage to the structures of PEM fuel cell components, including membrane, catalyst layer (CL) and gas diffusion layer (GDL).^[^ ^23^, ^24^, ^25^ ^]^ During the cold start, the ice formation, freeze–thaw cycle, and voltage reversal mainly cause the irreversible degradation of PEM fuel cell. Many experiments have been carried out to investigate the degradation phenomena during the cold start. Park et al. summarized the GDL degradation under the freeze‐thaw condition, including mechanical degradation and chemical degradation.^[^ ^26^ ^]^ Tabe et al. used a cryo‐scanning electron microscope to observe the cross‐sectional CL after the cold start and found that the region with ice formation moves from the membrane to the GDL during the freezing period.^[^ ^27^ ^]^ A simplified accelerated process with a PEM single‐cell was used to reveal the irreversible changes of the cathode during the repeated freeze cycles by Sabawa et al.^[^ ^28^ ^]^ Ozden et al. studied the degradation caused by the ice formation using the electrode specimens immersed in the deionized water under the continuous freeze–thaw cycles and found that the structural degradation of electrodes mainly depends on the ionomer type instead of the Pt loading.^[^ ^29^ ^]^ Zhong et al. studied the low temperature durability of a stack through segmented fuel cell and confirmed that the structure of electrodes is damaged due to the ice stress, resulting in the detachment and agglomeration of catalysts and even the delamination of CLs.^[^ ^30^ ^]^ However, these experiments adopted the ex‐situ test technique or in‐situ accelerated test technique, which is different from the real operating conditions.^[^ ^31^ ^]^ Moreover, the understandings of the underlying degradation mechanisms are scarce, due to the lack of comprehensive experimental techniques.

Therefore, it is still necessary to develop the comprehensive experimental techniques to reveal the underlying degradation mechanisms and summarize which degradation during the cold start is acceptable. In this study, the comprehensive in situ non‐accelerated techniques, including electrochemical performance test, electrochemical surface area (ECSA) degradation test, current and temperature distribution measurements, and pore characterization, are developed to analyze the degradation mechanisms under the conditions of freeze‐thaw cycle, voltage reversal, and ice formation. By analyzing the degradation caused by different factors, we summarize the degradation mechanisms into the degradation mechanism map in cold start of PEM fuel cell. This map demonstrates whether the ice formation is acceptable under the actual operating conditions and how to suppress the ice formation, and helps researchers understand the degradation mechanisms and develop the starting strategy to migrate the degradation.

## 2 Results and Discussion

**Figure 1:**
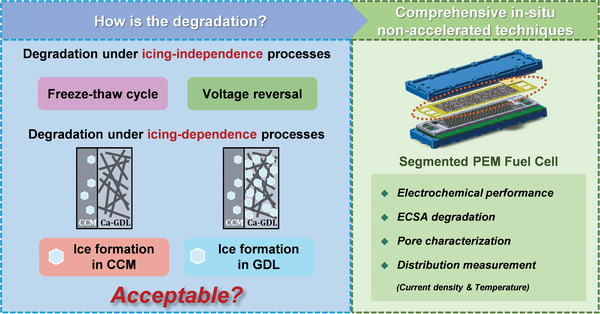
Schematic diagram of the factors that affect the degradation and the in‐situ non‐accelerated test technique.

The icing‐independence processes, including the freeze–thaw cycle and voltage reversal, and the icing‐dependence processes, including the ice formation in catalyst coated membrane (CCM) and GDL, are studied using the in situ non‐accelerated segmented fuel cell techniques in this study (**Figure** **1**). The same three procedures of conditioning, purging, and cooling down are first carried out before every cycle for all the processes, to ensure the same initial conditions of PEM fuel cells. **Table** **1** lists the startup conditions of different processes, including the freeze–thaw cycle, voltage reversal, and icing in CCM and GDL. During the test of freeze–thaw cycle, the open‐circuit fuel cell is directly heated up quickly using the auxiliary heating device. The voltage reversal was observed when the fuel cell starts with the constant current (CC) strategy in our previous work.^[^ ^32^ ^]^ Therefore, during the test of voltage reversal, the fuel cell first starts with the CC strategy and then artificially shuts down when the voltage reversal is observed (≈6 s). It is noteworthy that the water is not produced in the PEM fuel cell via the electrochemical reaction in this process.

**Table 1:** Startup conditions of different processes

	Freeze–thaw cycle	Voltage reversal	Short‐time icing in CCM	Long‐time icing in CCM	Short‐time icing in GDL	Long‐time icing in GDL
Ambient temperature [°C]	−15	−15	−15		−10	
Mass flow rates of gases (slpm)	–	0.56 of dry H_2_ in the anode and 2.27 of dry Air in the cathode				
Starting current density or voltage	–	0.5 A cm^−2^	0.45 V		0.3 V	
Voltage reversal	–	<6 s	–	–	–	–
Freezing time	–	–	–	9900 s	180 s	9900 s
Heating up	✓	✓	✓	✓	✓	✓

Furthermore, the degradation caused by ice formation is explored when the electrochemical reaction occurs in the PEM fuel cell. Based on our previous work, two cold start strategies are designed to form the severe icing in CCM and GDL, respectively, to investigate the degradation.^[^ ^32^ ^]^ The constant voltage (CV) strategy is employed to avoid the voltage reversal in these processes. The first strategy is to make the cold start quickly fail to ensure that the produced water only freezes in CCM and cannot flow into GDL. Then the PEM fuel cell is directly heated up above 0 °C using the auxiliary heating device, or continuously freezes for 9900 s and then is heated up, to investigate the degradation caused by the icing in CCM for different freezing time. To achieve the ice formation in GDL, the second strategy is to make the PEM fuel cell work for a period to ensure that the produced water can flow into GDL. It can be judged whether the water flows into GDL based on the cathode pressure drop (CPD).^[^ ^32^ ^]^ Then the PEM fuel cell is frozen for 180 or 9900 s, followed by the heating up, to investigate the degradation caused by the icing in GDL for different freezing time. Based on our previous work, the PEM fuel cell is frozen for 180 s to ensure that ice forms in the GDL.^[^ ^32^ ^]^ At this time, the order of magnitude of the 180 s is at 10^2^ s. In order for the ice stress to be fully effective, we set the PEM fuel cell to freeze for 10^4^ s. Considering the convenience of timing, we choose 9900 s as the freezing time, i.e., 2 h and 45 min. Finally, the strategy to suppress the ice formation and achieve the cold start without degradation is discussed, and the degradation mechanisms are summarized to guide the starting strategy development.

More experimental details are provided in the Supporting Information, including Figures S1–S4 and Table S1 (Supporting Information). Every cold start process is repeated for ten times to observe the degradation phenomena. ECSA is measured after cycles 1, 4, 7, and 10, respectively, to evaluate the degradation degree of PEM fuel cells. It is worth to note that the ECSA after the cycle 1 is adopted as the initial value to eliminate the possible errors caused by the individual difference of the same batch of commercial membrane electrode assembly (MEA). The measurements of the electrochemical performance and the distributions of temperature and current density are carried out after every cycle, while that of pore size distribution is carried out only after the last cycle.

### 2.1 Degradation under Freeze–Thaw Cycle Condition

**Figure 2:**
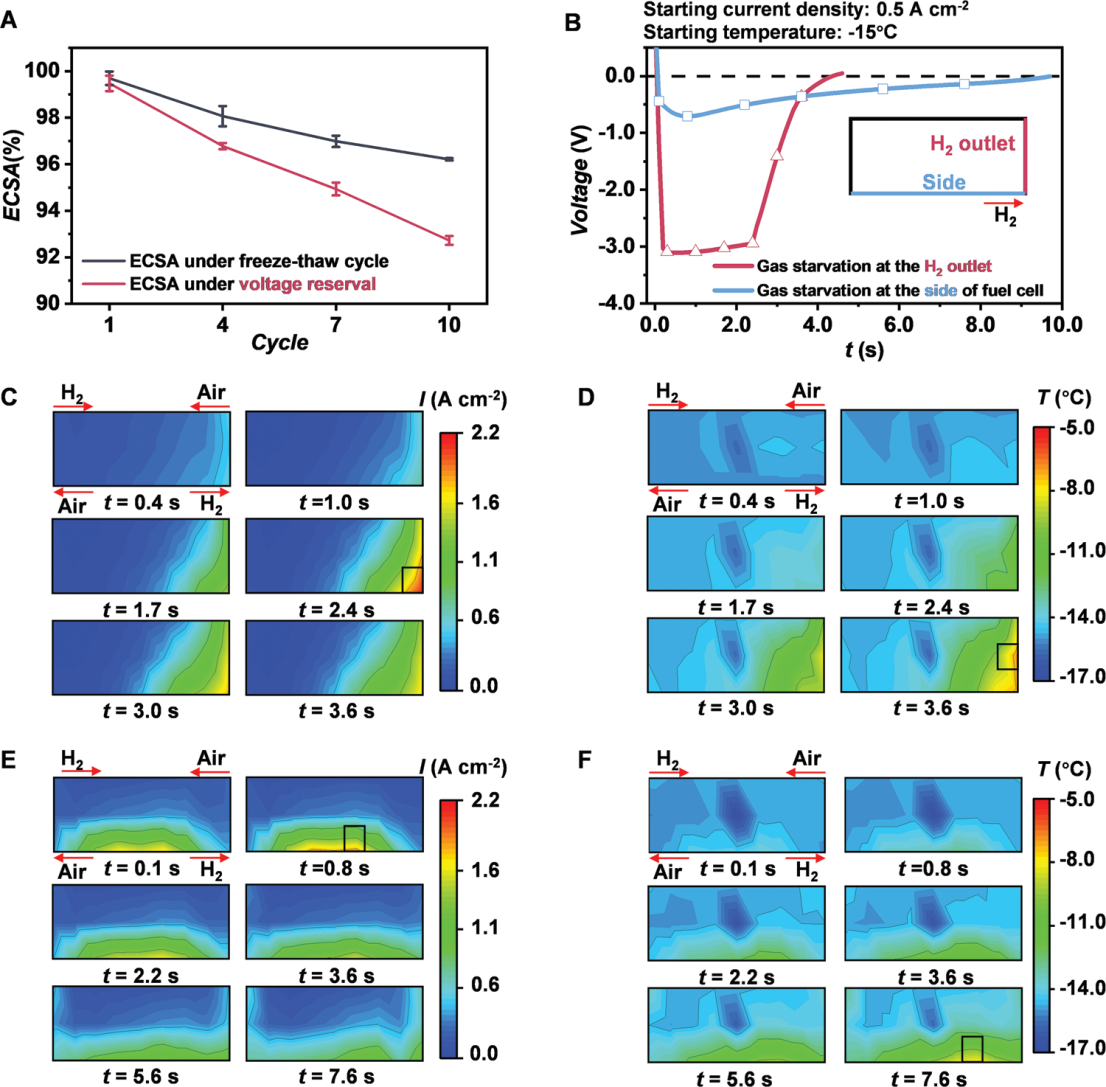
A) ECSA degradation caused by the freeze–thaw cycle and voltage reversal. B) Voltage changes of the MEAs with the gas starvation at the H_2_ outlet and the side of fuel cell, respectively. C) Current density distribution and D) temperature distribution for the MEAs with the gas starvation at the H_2_ outlet. E) Current density distribution and F) temperature distribution for the MEAs with the gas starvation at the side.

Freeze–thaw cycle is an inevitable process in the cold start of PEM fuel cell. Therefore, the degradation caused by freeze–thaw cycle is first investigated. The black line in **Figure** **2**A shows the ECSA every three cycles under the freeze–thaw cycle condition. The ECSA gradually decreases due to the degradation caused by the freeze–thaw cycle as shown in Figure 2A. The decreasing percents of ECSA are 1.63%, 1.08%, and 0.76% after the cycles 4, 7, and 10, respectively, which indicates that the degradation gradually becomes weaker. Physically, the mechanical structure of MEA gradually becomes stable during the cycles of thermal expansion and contraction, so the degradation gradually weakens. Therefore, the degradation caused by the freeze–thaw cycle is inevitable but acceptable.

### 2.2 Degradation Under Voltage Reversal Condition

Generally, the cold start with the CC strategy is better than that with the CV strategy. However, the voltage reversal easily occurs in the first few seconds when starting with the CC strategy. Therefore, the degradation caused by voltage reversal is investigated in this subsection. As shown in Figure 2A (pink line), the ECSA degradation under the voltage reversal condition is more severe than that under the freeze–thaw cycle condition and increases linearly with the increasing cycles. The pink curve in Figure 2B shows the voltage change during the cold start, and Figure 2C,D shows the current density and temperature distributions, respectively. It can be seen that the voltage reversal occurs at the H_2_ outlet, where the current density is high and a lot of heat is generated in a short time due to the voltage reversal. The highest current density and temperature raise to 2.13 A cm^−2^ and −6.81 °C, respectively, as shown in the black boxes in Figure 2C,D. The voltage reversal can be ascribed to be the gas starvation at the H_2_ outlet.

Interestingly, the voltage reversal is also observed at the side of PEM fuel cell in few experiments, as shown in Figure 2E,F. The highest current density and temperature are 1.87 A cm^−2^ and −7.94 °C, respectively, as shown in the black boxes in Figure 2E,F. As shown in Figure 2B, the voltage change (blue line) is different from the one (pink line) that the voltage reversal occurs at the H_2_ outlet, despite the same operating condition. It means that the gas starvation can possibly occur at the side of PEM fuel cell besides at the H_2_ outlet. Although the voltage reversal can provide a lot of heat for the cold start, it should be avoided due to the severe ECSA degradation. In future, developing the novel catalysts with a high tolerance of voltage reversal can help reduce the degradation.^[^ ^33^, ^34^ ^]^

### 2.3 Degradation Under Icing Condition in CCM

**Figure 3:**
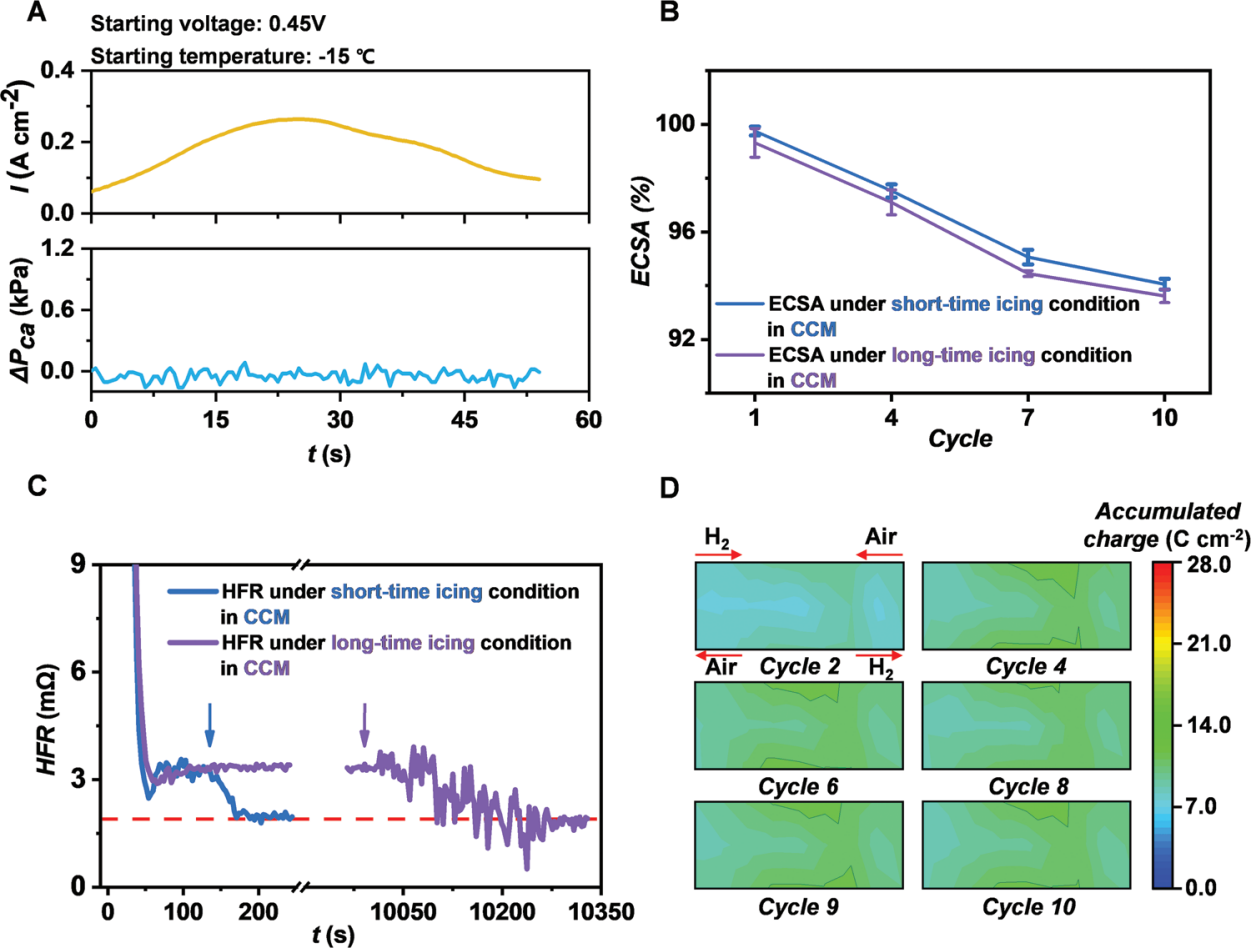
A) Output current density and cathode pressure drop when the PEM fuel cell starting at −15 °C. B) ECSA degradation under the short‐time and long‐time icing conditions in CCM. C) HFR change under the short‐time and long‐time icing conditions in CCM. D) Accumulated charge distribution after every cycle, which represents the distribution of produced water in CCM.

A lot of ice formation will not only lead to the failure of cold start but also cause the serious degradation of PEM fuel cell. The water is generated in the CCM when the electrochemical reaction occurs. Therefore, the icing in CCM is explored to know whether it is acceptable during the cold start. We also consider the long‐time icing in CCM when the PEM fuel cell is frozen at a sub‐freezing temperature environment for a long time. The PEM fuel cell starts with a CV strategy at 0.45 V from −15 °C, which leads to a quick failure of cold start due to the severe icing in CCM. One PEM fuel cell is directly heated up after failure, while another one continues to be frozen for 9900 s before heating up. **Figure** **3**A shows the output current density and CPD during the cold start. The current density first increases and then decreases with a stable CPD, meaning that the produced water is only in CCM and does not flow into GDL. Figure S5 (Supporting Information) shows the current density and temperature distributions during the cold start. The high‐current spot moves from the air inlet to the air outlet and the current density gradually decreases during the cold start, which means that the CCM is gradually hydrated by the produced water and then the produced water is frozen.

Figure 3B shows the ECSA degradation under the short‐time (blue line) and long‐time icing condition in CCM (purple line). The ECSA under the long‐time icing condition has a more severe degradation in comparison with that under the short‐time icing condition. The decrease of ECSA becomes slower, similar to that under the freeze–thaw cycle condition, because the structure of CCM becomes stable with increasing cycles. This cycle process of the icing in CCM also contains the freeze–thaw cycle process due to the continuous heating and cooling. Therefore, the ECSA degradations under the icing condition in CCM and the freeze–thaw cycle condition are compared in Figure S6 (Supporting Information). It can be found that the ECSA degradation under the icing condition in CCM is more severe, which means that the ice formation in CCM leads to the ECSA degradation except for the freeze–thaw cycle.

The hydration of electrolyte and membrane in CCM can be represented by the high frequency resistance (HFR) value as shown in Figure 3C, and the complete HFR change under the long‐time icing in CCM is shown in Figure S7 (Supporting Information).^[^ ^35^, ^36^ ^]^ The HFR is appropriately 1.9 mΩ when the electrolyte and membrane are sufficiently wetted, while it is higher than 1.9 mΩ when the fuel cell is frozen, which means that the ice formation will affect the proton transport in CCM. The HFR values under the short‐time icing and long‐time icing condition are nearly the same, which indicates that long‐time icing does not lead to more ice formation than short‐time icing. Therefore, the more severe degradation under the long‐time icing condition in CCM is ascribed to the long presence of ice instead of more ice.

In addition, the heating time under the long‐time icing condition in CCM is longer than that under the short‐time icing condition in CCM, as shown in Figure 3C. After long‐time freezing, the water in membrane tends to be in a critical state between ice and water due to the poor heat conductivity caused by the porous carbon fiber structure of GDL, thereby leading to the fluctuant HFR values during heating as shown in Figure 3C. However, the PEM fuel cell under the short‐time icing condition in CCM still has the residual heat because the electrochemical reaction just finished, leading to the faster ice melting (≈50 s) when heating. Therefore, the PEM fuel cell should be heated up quickly when failing in the cold start to protect it from the severe degradation.

Furthermore, the amount of charge produced by electrochemical reaction can represent the amount of produced water. We collect the amount of accumulated charge in the 27 segments of fuel cell after every cycle, as shown in Figure 3D. The accumulated charge distribution is nearly uniform, meaning that the amount of produced water in each segment is almost the same. Therefore, the same amount of ice frozen from the produced water possibly causes the same degradation, i.e., the degradation degrees of different regions of PEM fuel cell have little difference.

### 2.4 Degradation Under Severe Icing in GDL

**Figure 4:**
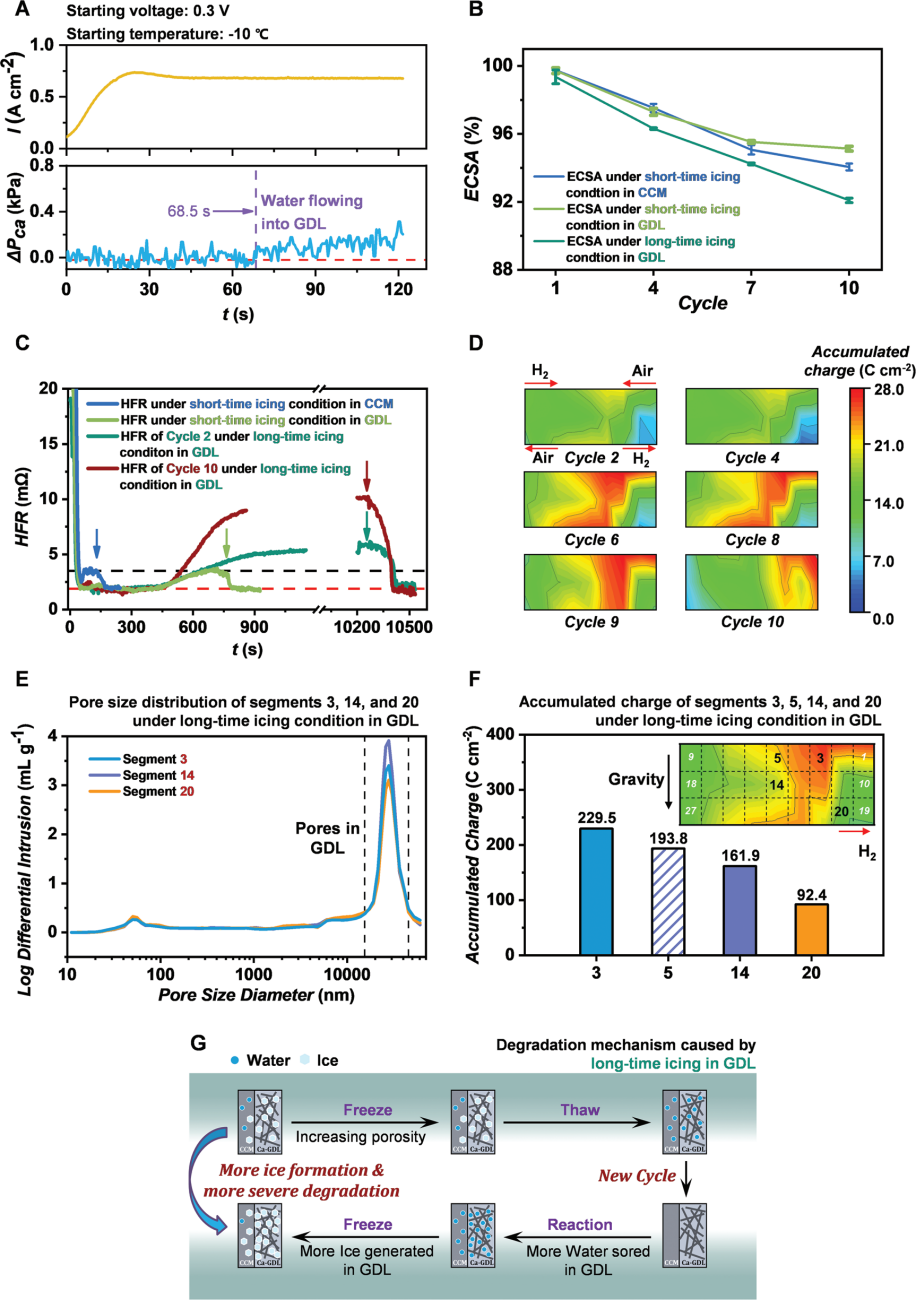
A) Output current density and cathode pressure drop when the fuel cell starting at −10 °C; B) ECSA degradation under the short‐time icing condition in CCM and the short‐time and long‐time icing conditions in GDL; C) HFR change under the short‐time icing conditions in CCM and GDL, as well as that for the cycles 2 and 10 under the long‐time icing condition in GDL. It is noteworthy that HFR change at different cycles is the same for the short‐time icing conditions in CCM and GDL. D) Accumulated charge distribution after ten cycles, which represents the distribution of produced water. E) Pore size distribution of segments 3, 14, and 20 under the long‐time icing condition in GDL after ten cycles, where the peak is related to the pores in GDL. F) Total accumulated charges of segments 3, 5, 14, and 20 after ten cycles and the location of different segments. G) Degradation mechanism caused by long‐time icing in GDL. Long‐time freezing increases the porosity of GDL due to the ice stress, which leads to more ice formation in next cycle and thus more severe ECSA degradation.

In order to investigate the icing in GDL, the PEM fuel cell starts with a CV of 0.3 V at −10 °C, to ensure that the produced water can flow from CCM into GDL. As shown in **Figure** **4**A, the CPD increases after 68.5 s, meaning that the water flows into GDL.^[^ ^32^ ^]^ Then the PEM fuel cells are frozen for 180 and 9900 s, respectively, to investigate the effects of short‐time and long‐time icing in GDL. As shown in Figure 4B, the ECSA degradation degree caused by short‐time icing in GDL is lower than that caused by short‐time icing in CCM. However, the long‐time freezing in GDL leads to the most severe ECSA degradation as shown in Figure S6 (Supporting Innformation).

The HFR values of different conditions are measured to further understand the underlying mechanism. The comparison of HFR values under the short‐time icing conditions in CCM (blue line) and GDL (light green line) is shown in Figure 4C. The minimum HFR in Figure 4C (red dot line), which is the same as the red dot line in Figure 3C, means that the electrolyte and membrane are well wetted. The same maximum HFR (black dot line) means that the amounts of ice in the membrane of PEM fuel cells are nearly the same. However, the PEM fuel cell under the icing condition in GDL takes a longer time (200 s) than that under the icing condition in CCM to reach the same icing state. The water in GDL preferentially freezes compared with that in CCM because the CCM is in the middle of PEM fuel cell. Therefore, the water in GDL can slow down the icing process of CCM and thus decrease the ECSA degradation to some degree. Moreover, the higher HFR value under the long‐time icing condition in GDL means the more ice formed in the membrane, compared with that under the short‐time icing condition in GDL as shown in Figure 4C. Additionally, the HFR value and its growing rate gradually increases with increasing cycles as shown in Figure 4C, which means that the icing gradually becomes more and quicker under the long‐time icing condition in GDL. The complete HFR change of the freezing and warming processes and the comparison of HFR in different cycles under the long‐time icing condition in GDL are shown in Figures S7 and S8 (Supporting Information), respectively.

The accumulated charges in each segment of the fuel cell with icing in GDL are counted and their distributions are shown in Figure 4D. Different from that under the icing condition in CCM, the accumulated charges in different segments under the icing condition in GDL are different, which means that the distribution of water is heterogeneous, thereby leading to the heterogeneous icing. The segments 3, 14, and 20 are cut out after ten cycles to investigate the effect of icing on the pore distribution. It is noteworthy that the amounts of accumulated charges at segments 3, 14, and 20 are from the high to low level. As shown in Figure 4E, the location with the peaks of pore distribution represents the pores in GDL.^[^ ^25^ ^]^ Therefore, the porosity in segment 14 is the largest, while that in segment 20 is least. To furthermore analyze the underlying mechanism, the accumulated charges of segment 5, that is located above the segment 14, and segments 3, 14, and 20 are compared as shown in Figure 4F. The accumulated charges of other segments are shown in Figure S9 (Supporting Information). As shown in Figure 4F, it can be inferred that the liquid water in GDL migrates vertically from the segments 5 to 14 because of gravity, thereby leading to the most water in the segment 14 among segments 3, 14, and 20. Therefore, more local water (i.e., more local ice formation) will increase the local porosity of GDL due to the ice stress.

The degradation mechanism caused by long‐time icing in GDL is summarized and shown in Figure 4G. Long‐time freezing increases the porosity of GDL due to the ice stress, which will store more water and produce more ice in GDL. Therefore, under the long‐time freezing, the ECSA degradation becomes more and more severe with increasing cycles. Although the water in GDL can slow down the icing process in CCM and decrease the ECSA degradation, the long‐time freezing will damage the GDL structure and thus result in more severe degradation, which should be avoided during the cold start of PEM fuel cells.

### 2.5 Starting Strategy to Suppress the Ice Formation

**Figure 5:**
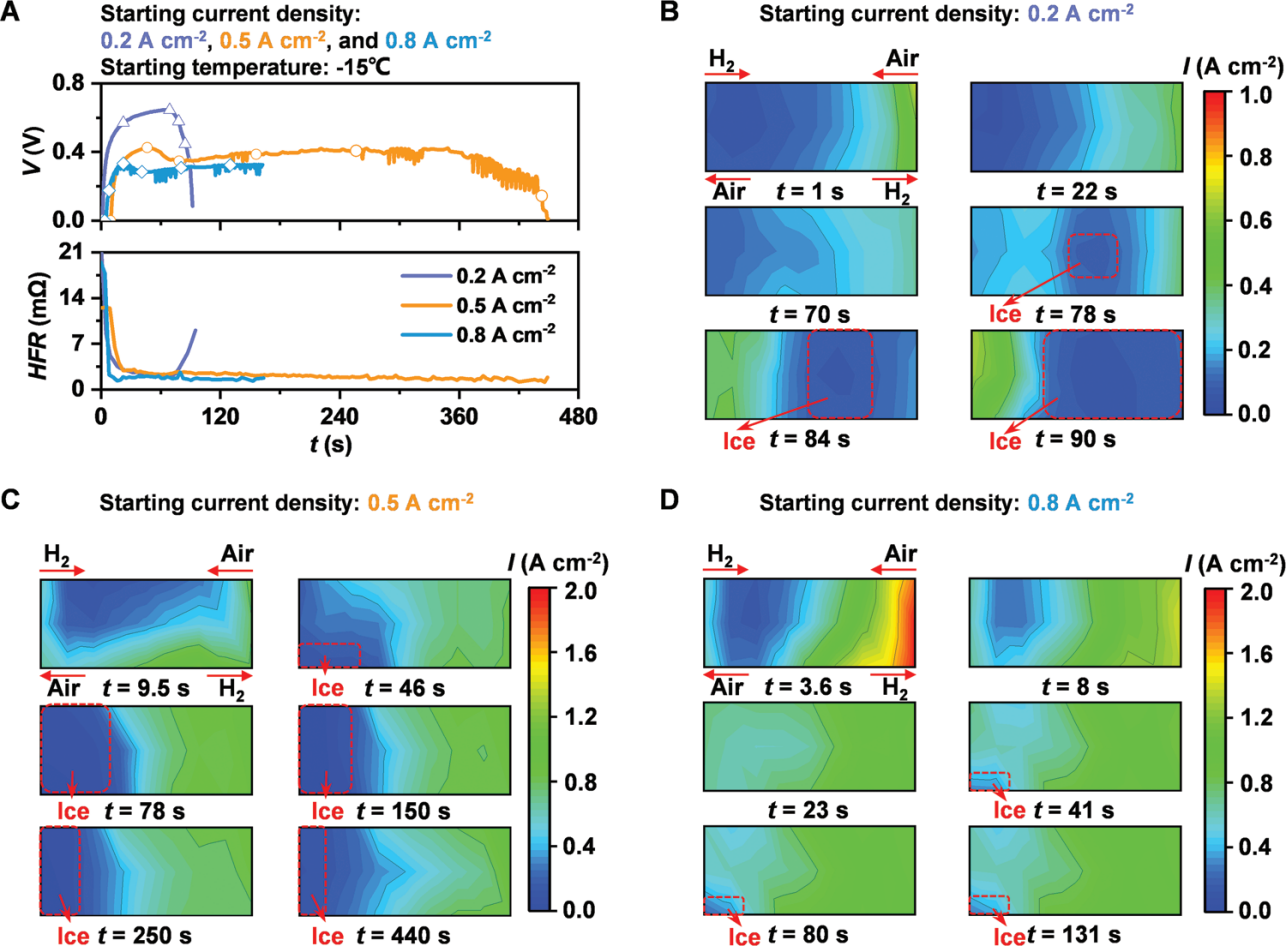
A) Output performance and HFR during the starting processes of PEM fuel cells with the starting current densities of 0.2, 0.5, and 0.8 A cm^−2^ at −15 °C. B–D) Current density distributions of PEM fuel cells starting with the starting current densities of B) 0.2 A cm^−2^, C) 0.5 A cm^−2^, and D) 0.8 A cm^−2^ during the cold start.

Based on the above analyses, the ice formation will significantly lead to the degradation of PEM fuel cells. Therefore, suppressing the ice formation through developing the starting strategy is necessary during the cold start of PEM fuel cells. In this subsection, the ice formation phenomena under the different starting current densities, including 0.2, 0.5, and 0.8 A cm^−2^, are investigated. **Figure** **5**A shows the changes of output voltage and HFR during the cold start. As the starting current density increases, the starting processes are a quick failure process caused by ice formation (0.2 A cm^−2^), a failure process caused by flooding (0.5 A cm^−2^), and a success process with local ice formation (0.8 A cm^−2^), respectively. Figure 5B–D shows the current density distribution during the cold start, where the low local current density represents the local ice formation as shown in the red box. The area of local ice formation location decreases significantly with increasing starting current density. The ice formation is the main factor that causes the degradation during the cold start. Therefore, without the voltage reverse, a higher starting current density is preferred to achieve a quick startup and suppress the ice formation, thereby decreasing the degradation during the cold start of PEM fuel cells.

### 2.6 Degradation Mechanism Map in Cold Start

**Figure 6:**
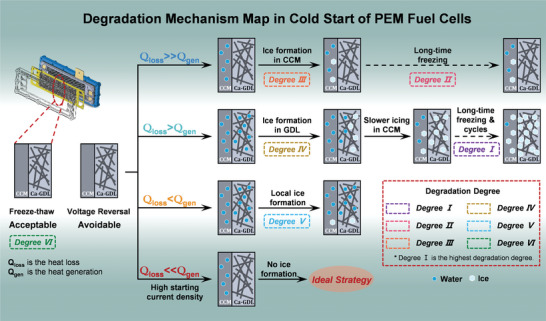
Degradation mechanism map in the actual cold start conditions of PEM fuel cells.

The effects of freeze–thaw cycle, voltage reversal, and ice formation on the degradation of PEM fuel cells are investigated in this study. The degradation mechanism map considering different processes is shown in **Figure** **6**, and the actual operating conditions corresponding to the different processes are listed in Table S2 (Supporting Information). The degradation degree is defined from degree I to degree VI, where degree I is the highest degradation degree and degree VI is the lowest one. The degradation degree caused by the freeze–thaw cycle is lower because of no ice formation, which is acceptable. The degradation caused by the voltage reversal can be avoided using the novel catalysts with a high voltage reversal tolerance. Furthermore, the ice formation in different components of PEM fuel cells with different freezing time will lead to different degrees of degradation. When the heat generation is much less than the heat loss, the produced water is quickly frozen in CCM that will lead to the degradation of PEM fuel cells, especially under the long‐time freezing. When the produced water can flow into GDL during the cold start, it will slow down the icing process in CCM and thus decrease the degradation to some degree. However, the long‐time freezing of water in GDL will damage the GDL structure and lead to more and more severe degradation as the number of startups increases. Therefore, quickly heating the PEM fuel cell after the failure of cold start is necessary to decrease the degradation. As the heat generation increases through increasing starting current density, the ice formation in PEM fuel cells can be effectively inhibited, thereby decreasing the degradation during the cold start. The ideal strategy is starting with a higher current density to obtain a quick startup without ice formation, thereby achieving the ideal cold start without degradation.

The degradation mechanism map proposed in this study is highly valuable. First, this map can help researchers understand the underlying degradation mechanisms in various processes. Furthermore, this map can also help develop the starting strategy with the low experiment and time cost. For example, the linear degradation, i.e., degree I shown in Figure 6, should be avoided during the cold start. In other words, the water in GDL should be purged after shutdown to avoid the damage to GDL structure due to the long‐time freezing. Moreover, the novel catalysts with a high voltage reversal tolerance should be developed in future to avoid the voltage reversal, especially at the H_2_ outlet. A higher staring current density is preferred to quickly warm the PEM fuel cells without ice formation and achieve the ideal cold start without degradation.

## 3 Conclusion

In this study, we investigated the degradation mechanisms under the freeze–thaw cycle, voltage reversal, and ice formation in CCM and GDL using in situ non‐accelerated test techniques, including electrochemical performance test, ECSA degradation test, pore characterization, and current density/temperature distribution measurement. Based on the experimental results, the degradation under the freeze–thaw cycle is slight and acceptable, while that under the voltage reversal is avoidable using the novel catalysts with a high voltage reversal tolerance. Moreover, the ice formation in different components of PEM fuel cells for different freezing time will lead to different degradation degrees. The long‐time icing in CCM will lead to a more severe degradation than the short‐time icing due to the influence of long‐time ice stress, although the amount of ice is the same. The water flows into GDL when more water is generated in CCM, which tends to slow down the icing process in CCM and decrease the degradation to some degree. However, long‐time freezing of water in GDL damages the GDL structure and increases the porosity of GDL, which leads to more water stored in GDL and thus more severe degradation due to the more and quicker icing in the next cold start. Therefore, the ice formation is the major factor that causes the degradation during the cold start of PEM fuel cells. A higher starting current density is preferred to avoid the ice formation and achieve the ideal cold start without degradation. Degradation mechanism map in the cold start of PEM fuel cells is summarized in this paper, which can help researchers understand the underlying degradation mechanisms in various processes and develop the cold start strategy.

## Conflict of Interest

The authors declare no conflict of interest.

## Author Contributions

J.L. and K.J. conceptualized the study; J.L., L.F., Q.D., Y.Y., and K.J. contributed to methodology; J.L. performed investigations; L.F. and K.J. acquired resources; J.L. and L.F. wrote the original draft; J.L., L.F., and K.J. reviewed and edited the final manuscript; K.J. supervised the study; L.F., Q.D., and Y.Y. acquired funding.

## Supporting information

Supporting Information

## Data Availability

The data that support the findings of this study are available in the supplementary material of this article.
