# Supplementary material for: Ice Formation during PEM Fuel Cell Cold Start: Acceptable or Not?
Source: Adv Sci (Weinh). 2023 Jun 21;10(24):2302151. doi: 10.1002/advs.202302151 (PMC10460847; doi:10.1002/advs.202302151)
Supplement: Supplementary file 1 — Supporting Information [file ADVS-10-2302151-s001.pdf]

ADVANCED SCIENCE
Open Access

Supporting Information

for *Adv. Sci.*, DOI 10.1002/advs.202302151

Ice Formation during PEM Fuel Cell Cold Start: Acceptable or Not?

Jinqiao Liang, Linhao Fan, Qing Du, Yan Yin and Kui Jiao**

Supporting Information

Ice Formation during PEM Fuel Cell Cold Start: Acceptable or not?

Jinqiao Liang, Linhao Fan, Qing Du, Yan Yin and Kui Jiao**

J.L., L.F., Q.D., Y.Y., K.J.

State Key Laboratory of Engines, Tianjin University, 135 Yaguan Road, Tianjin, 300350, China

National Industry-Education Platform of Energy Storage, Tianjin University, 135 Yaguan Road, Tianjin, 300350, China

E-mail: lhfan@tju.edu.cn, kjiao@tju.edu.cn

Segmented fuel cell design

In this study, the segmented fuel cell is used to measure the current density and temperature distributions of PEM fuel cells during cold start processes, as shown in Figure S1. It is necessary to replace the cathode collector and the bipolar plates in the traditional PEM fuel cell with the printed circuit board (PCB) and the insulated segmented bipolar plates, respectively, to obtain the corresponding segmented fuel cell as shown in Figure S1A. A 10 mΩ resistor in the PCB constitutes an electrical path with each segmented cathode bipolar plate as shown in Figure S1B. The current in each path, i.e., the current in each segment, can be measured by the voltage difference between the two ends of the resistor. The current density distribution is obtained by dividing the current by the area of every segment. In traditional PEM fuel cells, the temperature at a central point in the cathode is usually measured as the temperature of the whole cathode, as shown in Figure 1C. In the segmented PEM fuel cell developed by this study, temperatures are taken from 27 points in the cathode, each of which has an area of 4 cm^2. The reliability of the segmented fuel cell has been verified in our previous paper.[1]

Experimental system

The experimental system employed to study the cold start processes of PEM fuel cell primarily consists of five main components: a 1 kW fuel cell test station, an electrochemical workstation, an environmental chamber, a DAQ system, and an auxiliary heating device as shown in Figure S2. The 1 kW fuel cell test station (NBT-1000W, NBT) can measure the output current or voltage of the whole fuel cell through the load station, and control and record the parameters of the gases (air, H_2, and N_2) entering the fuel cell, including the mass flow rate, temperature, relative humidity (RH), and pressure. The electrochemical workstation (Zennium Pro.,

Zahner) can measure the HFR and other electrochemical diagnostics. The environmental chamber (H/GDW-50L, HuSheng Test Instrument) can provide a stable environmental temperature ranging from -50 ℃ to 200 ℃ with an error of less than 0.5 ℃. The DAQ system comprises the multi-channel voltage acquisition cards (NI-9205, National Instruments) and temperature acquisition cards (NI-9214, National Instruments). Labview is employed to control the DAQ program and record the distribution data with a frequency of 10 Hz. The auxiliary heating device (TLTP-TEC2410D, Wuhan Tailunte Century Technology Co., Ltd) is used to heat the PEM fuel cell from the ambient temperature to above 0 ℃.

Test procedures

To ensure the same initial conditions of PEM fuel cells, the same three procedures of conditioning, purging, and cooling down are firstly carried out before every cycle for all the processes. The parameters of operating conditions are listed in Table S1. PEM fuel cell operates for 0.5 h under the condition of 70 ℃, 0.4 V, and 1 atm in the conditioning procedure with the supplied 40% RH H_2 of 1.6 slpm and 40% RH Air of 6.4 slpm. Then PEM fuel cell is purged with the dry N_2 with a mass flow rate of 2 splm for anode and cathode at 25℃ during the purging procedure, and the HFR is measured at 1000 Hz. When the HFR raises to 11 mΩ, the purge gas flow is successively adjusted to 1 slpm, 0.5 slpm, and 0 slpm to ensure the sufficient dry environment inside PEM fuel cell and avoid the effect of water redistribution. The HFR is stable around 11 mΩ after purging. After the purging procedure, PEM fuel cell is frozen at the specific temperature for 6 h to ensure the same temperature in different regions of fuel cell by measuring the temperature distribution. The startup procedure of PEM fuel cell is carefully designed to study the conditions of freeze-thaw cycle, voltage reversal, and ice formation. Every startup process is repeated for 10 times to observe the degradation phenomena. Figure S3 shows the heating process of PEM fuel cell using the auxiliary heating device. It can be found that the auxiliary heating device can warm PEM fuel cell linearly and uniformly to avoid the experimental error. The linear sweep voltammetry curves shown in Figure S4 are nearly the same under different conditions, which indicates that the membrane is not damaged.

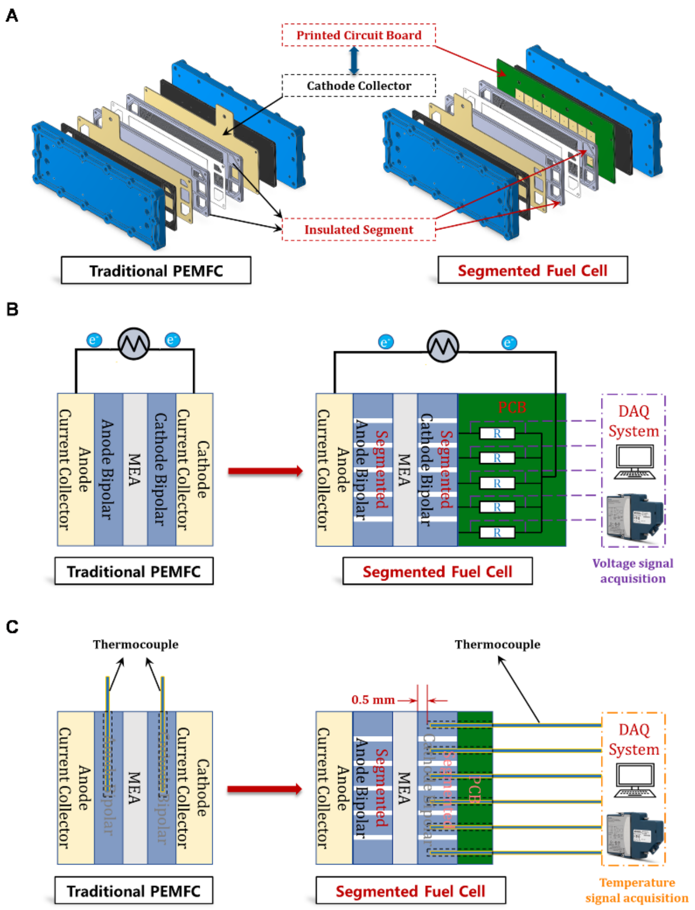

Figure S1. Schematic diagram of segmented fuel cell design. (A) Structures of the traditional PEM fuel cell and the segmented PEM fuel cell. (B) Principle of current density distribution measurement. (C) Principle of temperature distribution measurement.

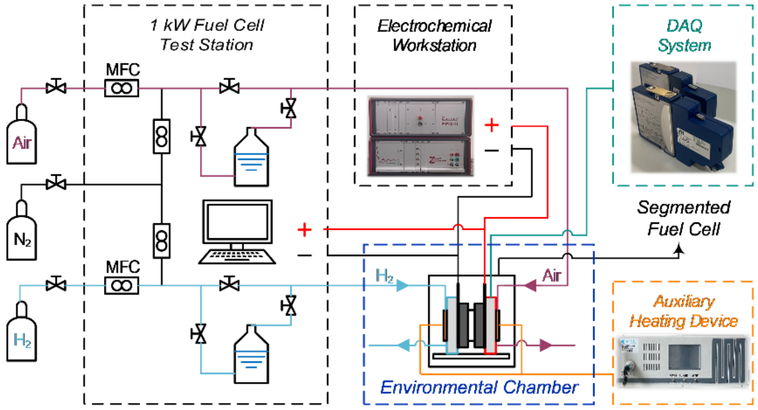

Figure S2. Schematic diagram of experiment system for the cold start of PEM fuel cell. The experiment system consists of five main equipment, including a 1 kW fuel cell test station, an electrochemical workstation, an environmental chamber, a DAQ system, and an auxiliary heating device.

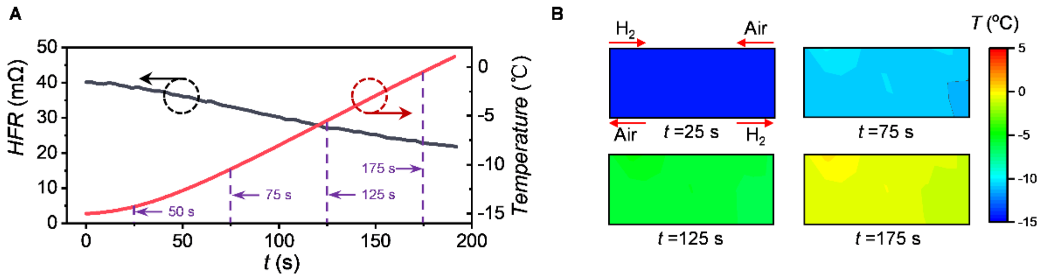

Figure S3. Heating process of PEM fuel cell. (A) HFR and temperature changes during the heating process. (B) Temperature distributions of PEM fuel cell during the heating process.

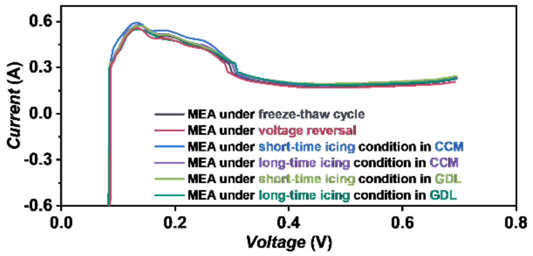

Figure S4. Linear sweep voltammetry curves under different operating conditions after 10 cycles.

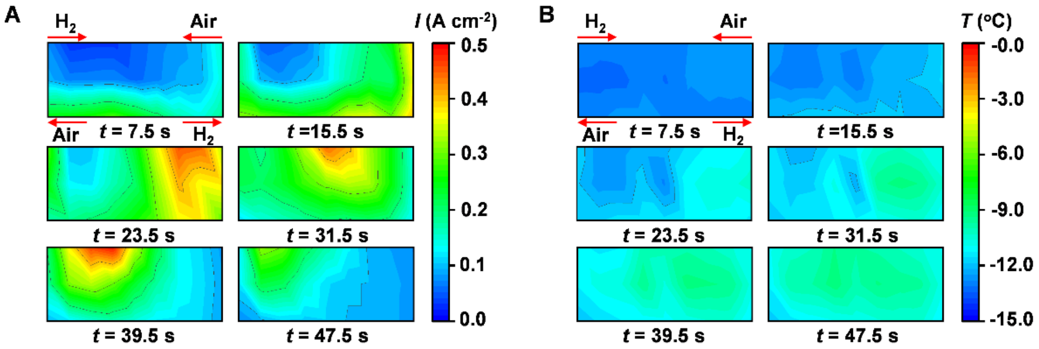

Figure S5. (A) Current density and (B) temperature distributions under the icing condition in CCM.

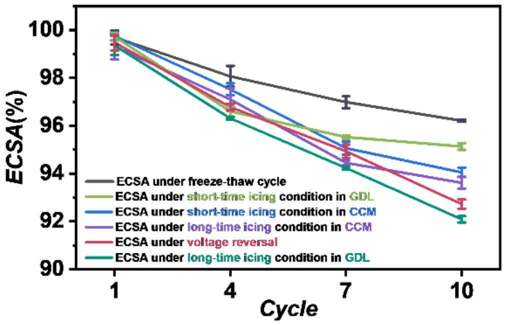

Figure S6. ECSA degradation of under different conditions.

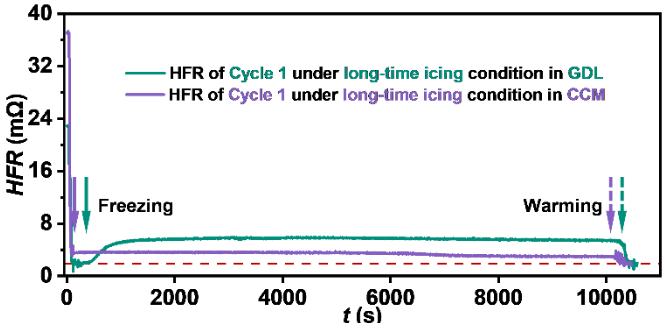

Figure S7. HFR change under the long-time icing conditions in GDL and CCM.

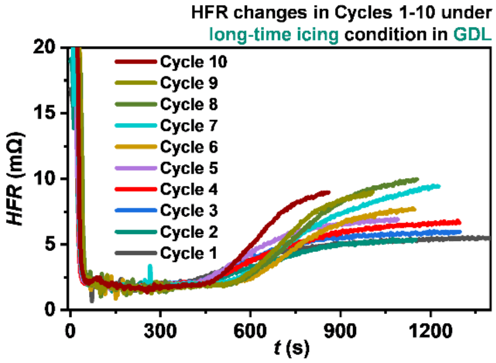

Figure S8. HFR changes in different cycles under the long-time icing condition in GDL.

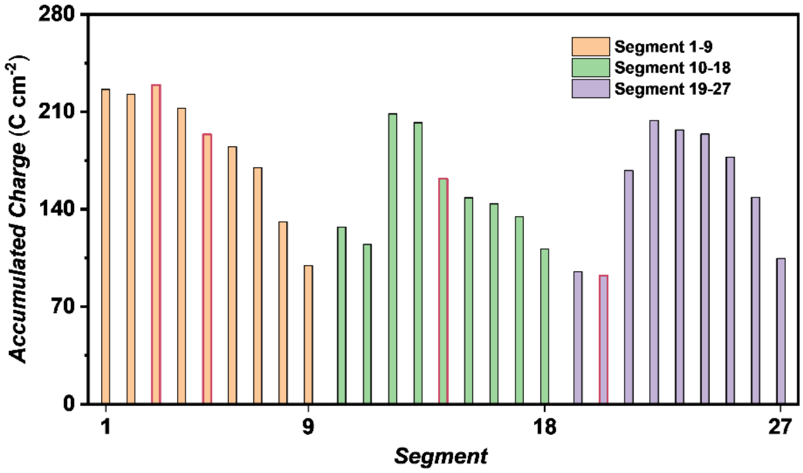

Figure S9. Accumulated charges in every segment under the long-time icing condition in GDL.

Table S1. The parameters of different operating conditions.

**Parameters**		**Value**
Conditioning	Temperature of supplied gases	70 °C
	Temperature of PEM fuel cell	70 °C
	Relative humidity of supplied gases	40%
	Absolute pressure at outlet	1 atm
	Voltage	0.4 V
	Duration	0.5 h
Purging	Temperature of N_2	25 °C
	Relative humidity of N_2	0%
	Absolute pressure at outlet	1 atm
Cooling down	Temperature of environment	-5, -10, and -15 °C
	Duration	6 h

Table S2. Actual operating conditions and degradation degrees under different processes.

Process	Reason	Actual operating condition	Degradation degree
Freeze-thaw cycle	-	Freezing and thawing during the cold start	Acceptable degradation
Voltage Reversal	Gas starvation	Usual occurrence when starting with constant current	Avoidable using novel catalysts
No ice formation	$Q_{loss} << Q_{gen}$	Ideal cold start	-
Local ice formation	$Q_{loss} < Q_{gen}$	Successful cold start with local ice formation	V
Short-time ice formation in GDL	$Q_{loss} > Q_{gen}$	Quickly heating fuel cell after failure of cold start	IV
Long-time ice formation in GDL		No artificial intervention after failure of cold start	I
Short-time ice formation in CCM	$Q_{loss} >> Q_{gen}$	Quickly heating fuel cell after quick failure of cold start	III
Long-time ice formation in CCM		No artificial intervention after quick failure of cold start	II

References

1. J. Liang, L. Fan, T. Miao, X. Xie, Z. Wang, X. Chen, Z. Gong, H. Zhai, K. Jiao, *J. Mater. Chem. A* **2022**, *10*, 20254.
